# Serpents Bite and Other Poisoned Wounds

**Published:** 1855-01

**Authors:** 


Serpents Bite and other Poisoned Wounds.—Dr. Brainard, of Chicago, in an interesting essay on "treating Serpent bite and other poisoned wounds," concludes with the following observations:—

The result of my experiments, taken in connection with those made by others, is, that up to the present time, no substance or solution has been found capable of preventing the fatal effects of the rattlesnake bite (unless destroying by caustic or excising the tissues of the part) excepting the solution of iodine. This, within certain limits, is capable of neutralizing it.

In order to guard against error, it should be stated that, in experimenting with birds, the separation of the skin by injecting even distilled water and applying cupping-glasses, is liable to cause the skin to fall off in scales. When the skin is not extensively separated, the solution recommended does not produce an eschar. The effects of iodine solutions on the tissues, since the publication of Velpeau's work on the subject, are so well understood that it is unnecessary to dwell further on this point.

The effect of solutions of iodine, infiltrated into the tissues around the bite of the rattlesnake, is to prevent their discoloration and preserve the natural texture and color of the parts. Even when it does not prevent death from occurring, it still has this effect in a great degree.

The deductions which I think may legitimately be made from the foregoing facts, are—

1. The venom of the crotalus produces spasm of all the muscles—most marked in the muscles of respiration.
2. This venom produces a peculiar change of the blood globules which consists of alteration of form and disintegration.
3. If death is delayed, it deprives the blood of its fibrine.
4. The solution of iodine and iodide of potassium, in the proportion of ten grains of the former and thirty of the latter to the ounce of distilled water, is, wi hin certain limits, an antidote to the venom of the rattlesnake.
5. When the venom is deeply inserted, or when it has been absorbed, the antidote, to be effectual, must be infiltrated into the tissues.
6. This infiltration can be performed without causing loss of substance, or producing either eschar or suppuration.

